# Role of universal health coverage in improving quality of breast cancer care: an international comparison study

**DOI:** 10.1136/bmjph-2023-000863

**Published:** 2024-09-24

**Authors:** Minmin Wang, Suhang Song, Yinzi Jin, Zhijie Zheng

**Affiliations:** 1 School of Public Health, Peking University, Beijing, China; 2 Institute for Global Health and Development, Peking University, Beijing, China; 3 Department of Health Policy and Management, University of Georgia, Athens, Georgia, USA

**Keywords:** Public Health, Epidemiology, Public Health Practice

## Abstract

**Introduction:**

Breast cancer is the most common and lethal cancer among women worldwide. Good quality cancer care is a key pillar in improving the survival rate and reducing the burden of this cancer. This study aimed to evaluate the current status and temporal trends in global breast cancer care and to identify the association between universal health coverage and quality of breast cancer care.

**Methods:**

A quality of care index for breast cancer was constructed using disease burden data from the Global Burden of Disease 2019 database. This index was evaluated and compared at global, regional and national level. The association between universal health coverage index and breast cancer quality of care index at national level was also explored.

**Results:**

The quality of breast cancer care improved from 1990 to 2019, and the disparity narrowed between countries at different development levels over the same period. The universal healthcare coverage index was positively associated with national breast cancer care quality. This finding was robust across countries at low and middle levels of development, as well as more developed countries.

**Conclusions:**

The identified association between universal health coverage and breast cancer care highlight the key role of developing a high-quality and resilient healthcare system for improving breast cancer care. Then expanding the universal health coverage with inclusion of breast cancer care may help improving the breast cancer care quality and reduce the disproportionate mortality due to breast cancer in low social development countries.

**WHAT IS ALREADY KNOWN ON THIS TOPIC**

**WHAT THIS STUDY ADDS**

- The quality of breast cancer care improved from 1990 to 2019, and the disparity narrowed between countries at different development levels over the same period.
- Universal healthcare coverage index was positively associated with breast cancer care quality.

**HOW THIS STUDY MIGHT AFFECT RESEARCH, PRACTICE OR POLICY**

- It is important to address the vulnerabilities in accessing good quality breast cancer care. Developing a high-quality and resilient healthcare system is the key strategy for improving breast cancer care.

## Introduction

 Breast cancer is the most prevalent malignancy worldwide, and also the leading cause of cancer-related mortality among women. According to estimates from the International Agency for Research on Cancer, in 2020, breast cancer accounted for a staggering 2 261 419 incident cases and claimed the lives of 684 996 individuals,^1^ rendering it the most common and fatal cancer in the global female population. The prognosis for breast cancer varies considerably across geographical regions, with high-income countries having 5-year survival rates surpassing 90%, and low-income countries such as South Africa having rates lower than 50%.^2^,^4^ Recognising the urgency of the situation, the WHO established the Global Breast Cancer Initiative in 2021.^5^ The primary objective of this initiative is to address the escalating breast cancer epidemic and to reduce breast cancer mortality each year by 2.5% over a span of 20 years, ultimately preserving 2.5 million lives.

It is well known that the level of care that patients receive significantly influences their prognosis. Accumulating evidence has highlighted the potential preventive and control measures for breast cancer. For instance, population-level breast cancer screening programmes using standard mammography are cost-effective in reducing both incidence and mortality of breast cancer.^6^,^11^ Novel treatment strategies have also contributed to improved breast cancer outcomes. These include combining surgery, chemotherapy, radiation therapy and endocrine therapy within a multidisciplinary treatment framework and precision treatment conception.^12^,^14^

It is possible that achieving better quality of care hinges on the resilience of healthcare systems and the attainment of universal health coverage (UHC). This study, therefore, aimed to assess the global, regional and national quality of breast cancer care, using a quality of care index (QCI) as an evaluative tool. We also aimed to investigate the association between UHC and the QCI for breast cancer. Our findings will enable a comparative analysis of breast cancer care quality worldwide and provide valuable evidence for the development of a global strategy for breast cancer prevention and control. In particular, it will highlight the role of UHC and a resilient healthcare system.

## Materials and methods

### Data sources

The burden of breast cancer was extracted from the Global Burden of Disease (GBD) 2019 dataset using the Global Health Data Exchange (https://ghdx.healthdata.org).^15^ GBD 2019 incorporated nationally representative surveys, censuses and meta-analysis results to estimate the incidence, prevalence, mortality, years of life lost (YLLs), years lived with disability (YLDs) and disability-adjusted life-years (DALYs) for 369 diseases and injuries in 204 countries and territories. The overall GBD 2019 methodologies have been detailed previously.^16^ We collected six indicators of breast cancer from the GBD 2019 dataset (incidence, prevalence, mortality, DALY, YLL and YLD).

The data on the national universal healthcare coverage index were extracted from the WHO Global Health Observatory (https://www.who.int/data/gho).^17^ Coverage of essential healthcare services was defined as the average coverage of essential services based on tracer interventions that include reproductive, maternal, newborn and child health, infectious diseases, non-communicable diseases and service capacity and access, among the general and the most disadvantaged populations. The indicator is an index reported on a unitless scale of 0–100, which is computed as the geometric mean of 14 tracer indicators of healthcare service coverage.

### Quality of care index

The QCI was constructed from the perspective of disease burden. First, four secondary indicators were constructed using age-standardised rates retrieved from the GBD 2019 dataset. These were (1) ratio of YLLs to YLDs, (2) ratio of DALYs to incidence, (3) mortality-to-incidence ratio and (4) prevalence-to-incidence ratio. Ratio of YLLs to YLDs, ratio of DALYs to incidence and mortality-to-incidence ratio were all considered to be negatively associated with the quality of care and prevalence-to-incidence ratio was positively associated with quality of care. The QCI was determined using principal component analysis (PCA) based on these four secondary indicators. PCA uses mathematical multivariable analysis to extract linear combinations as orthogonal components of the four secondary indicators. The first component that best describes the variance and variability in the data is the QCI and allocated a score of 0–100. Higher scores on the QCI indicate a higher quality of breast cancer care.

(1) Ratio of YLLs and YLDs =YLLs/YLDs

(2) Ratio of DALYS to incidence=DALYs/prevalence

(3) Mortality−to−incidence ratio=Mortality/Incidence

(4) Prevalence−to−incidence ratio=Prevalence/Incidence

### Statistical analysis

We analysed the disease burden and trends in breast cancer from 1990 to 2019. We constructed the QCI and examined the disparities in this index across regions, countries and age groups. Breast cancer QCI was also evaluated in each sociodevelopment quintile, defined using the Sociodemographic Index (SDI). The SDI is a geometric average of 0–1 in each country or region. It is obtained by combining the total fertility rate of women younger than 25 years, the education level of people aged 15 years and older, and the lag in the per capita income distribution. Countries and regions were divided into five levels by SDI: high (>0.81), high-middle (0.70–0.81), middle (0.61–0.69), low-middle (0.46–0.60) and low (<0.46).

To explore the relationship between UHC and quality of breast cancer care, we constructed a linear regression model with breast cancer QCI as the dependent variable and breast cancer screening coverage as the independent variable. We also examined the association separately in low and high development countries. Sensitivity analysis used the UHC service coverage index in 2000, 2010 and 2015 to test the robustness of the association.

All statistical analyses used STATA (V.14.0; StataCorp) and R V.4.1.3 software (http://www.r-project.org/). All tests were two sided and *P* values <0.05 were considered statistically significant.

## Results

### QCI for breast cancer

In 2019, the global average QCI for breast cancer care reached 75.45, reflecting an annual increase from 69.45 in 1990. Of the 21 regions defined by the GBD study, the high-income Asia Pacific region had the highest quality of breast cancer care, with a QCI of 96.58. The lowest quality of care was in Eastern sub-Saharan Africa, with a QCI of 13.04 (online supplemental table 1). Notably, from 1990 to 2019, the QCI for breast cancer care in Eastern Sub-Saharan Africa experienced a remarkable 6.32-fold increase, rising from 2.92 to 18.46. The QCI remained stable in the high-income Asia Pacific region, ranging from 95.76 to 96.58.

**Figure 1:**
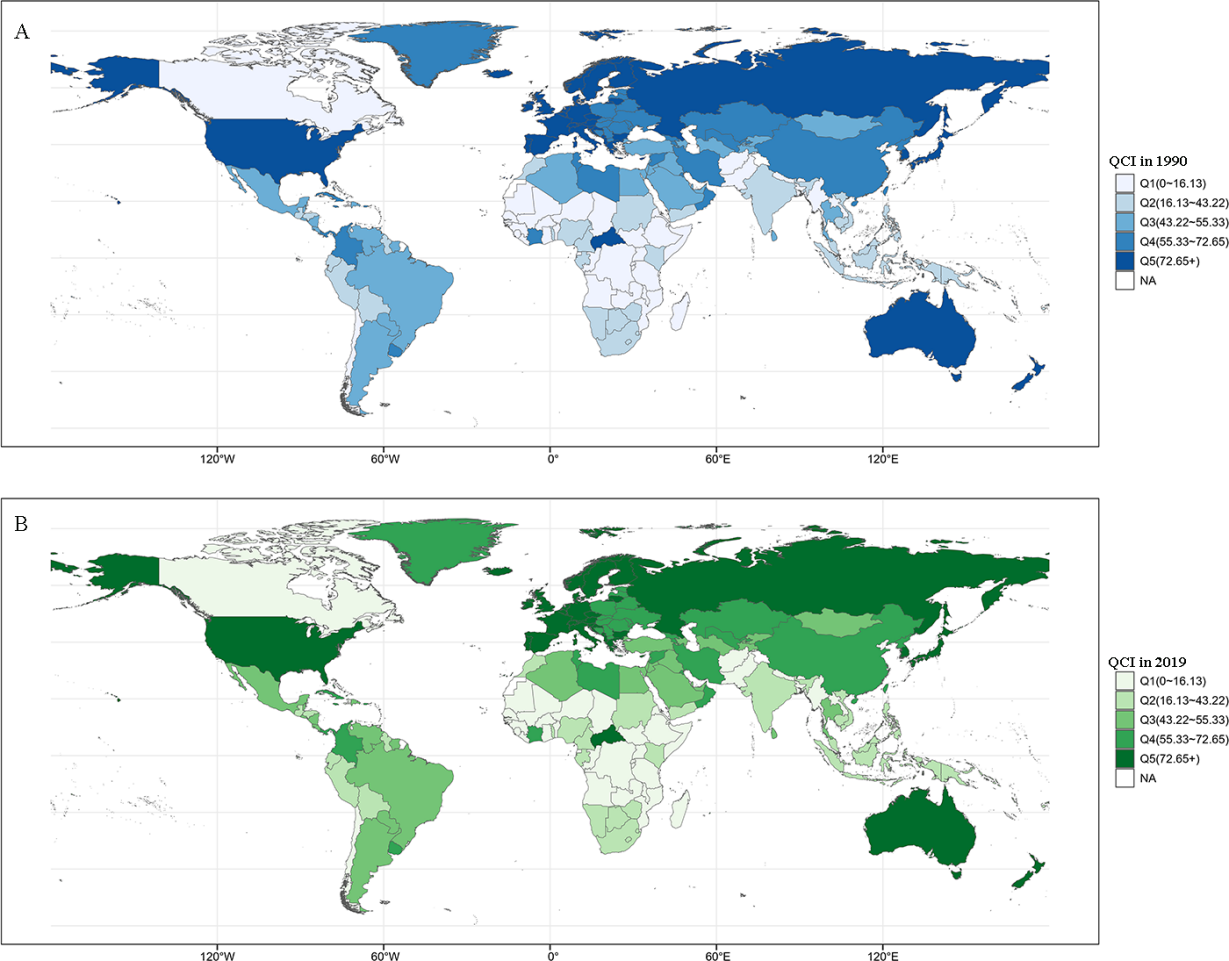
The global map of breast cancer quality of care index (QCI) in 1990 and 2019. The cut-offs were chosen by the five quintiles of QCI index in 1990.

There were also notable variations in the QCI for breast cancer care across different countries. Figure 1 shows the global distribution of the QCI for breast cancer care in 1990 and 2019. In 1990, Japan had the highest quality of care, with a QCI of 98.66, and Rwanda had the lowest QCI of 6.17×10^−4^. By 2019, Iceland had achieved the highest QCI (99.98), and the Central African Republic had the lowest (3.57).

**Figure 2:**
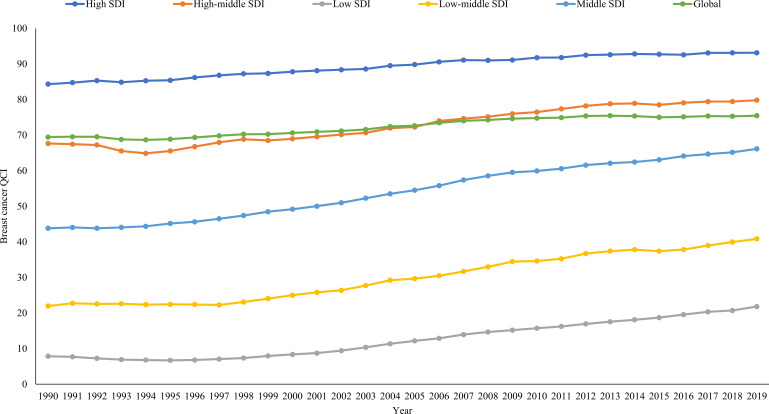
The temporal trends in breast cancer quality of care index (QCI) by Sociodemographic Index regions, from 1990 to 2019.

The QCI for breast cancer care showed a positive association with social development levels. It was 93.13, 79.81, 66.13, 40.86 and 21.83 in high, high-middle, middle, low-middle and low SDI regions. Over the period from 1990 to 2019, the QCI for breast cancer care consistently improved across all SDI regions, and the gaps in QCI between SDI regions gradually narrowed (figure 2).

QCI for breast cancer care showed a slightly increasing trend with advancing age, although the patterns differed across SDI regions. High, high-middle and middle SDI regions showed a parallel increasing, followed by a decline trend, with the peak QCI observed at ages 75–79 or 65–69 years. In contrast, the QCI for breast cancer care in low-middle and low SDI regions showed a fluctuating pattern (online supplemental figure 1).

### UHC and QCI for breast cancer care

**Figure 3:**
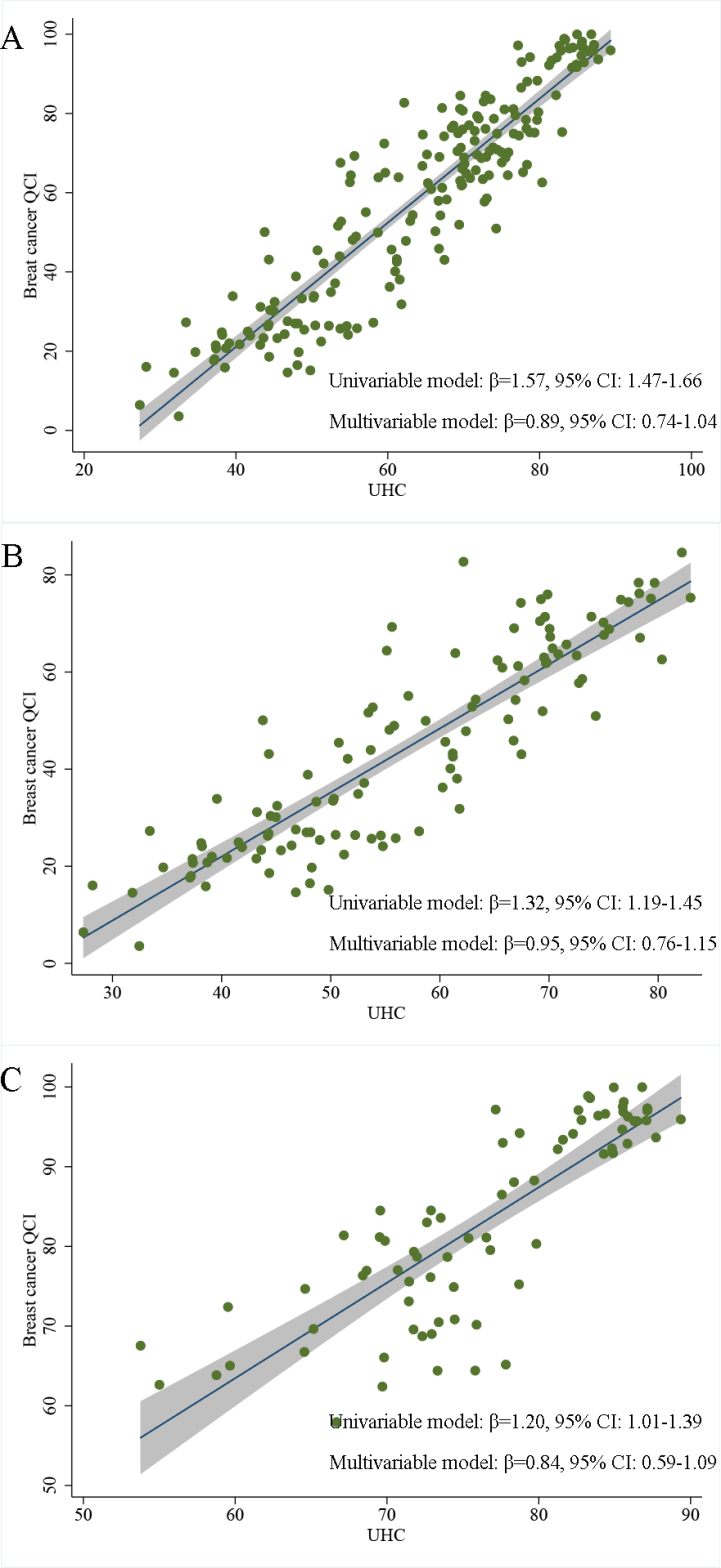
The association between universal healthcare coverage index and breast cancer quality of care index (QCI) at country level. (**A**) The association estimation in 194 countries included in this study; (**B**) The association estimation in low, low-middle and middle development regions (by Sociodemographic Index); (**C**) The association estimation in high and high-middle development regions (by Sociodemographic Index). UHC, universal health coverage.

The association between UHC and QCI for breast cancer care is shown in figure 3. Among the 194 countries with complete data on UHC index and QCI for breast cancer care, there was a positive association between the two. For each incremental increase in UHC, the QCI for breast cancer care increased by an average of 1.57 units (95% CI 1.47, 1.66). After adjusting for the SDI index as a covariate, the estimated effect size remained at 0.89 (95% CI 0.74, 1.04). In countries with low, low-middle and middle SDI, the β coefficient was 1.32 (95% CI 1.19, 1.45) in the univariable estimation and 0.95 (95% CI 0.76, 1.15) in the multivariable regression model. In countries with high and high-middle SDI, the effect sizes were 1.20 (95% CI 1.01, 1.39) in the univariable model, and 0.84 (95% CI 0.59, 1.09) in the multivariable model. Sensitivity analyses were robust: the UHC index in the years 2000, 2010 and 2015 was also positively associated with the country-level breast cancer QCI (online supplemental table 2).

## Discussion

Breast cancer has emerged as a critical global health challenge necessitating immediate attention and high-quality care was one of the key strategies.^18^ In this study, we used the QCI to comprehensively evaluate the quality of breast cancer care at a global, regional and national level. Our findings showed an overall enhancement in the quality of global breast cancer care from 1990 to 2019, accompanied by a diminishing disparity in QCI scores across countries at different levels of development. We also found a positive and independent association between the quality of breast cancer care and the country-level provision of universal healthcare services. This relationship remains robust across low-development and middle-development countries, as well as high-development countries. The outcomes of this study shed light on the present status of breast cancer care and underscore the potential mechanism for reducing the burden of breast cancer by augmenting universal health service coverage and expanding the capacity of healthcare systems.

Breast cancer care experienced overall improvement at global level while disparity across regions still raise concerns. Disproportionate mortality was observed that low SDI regions accounted around 4% of global breast cancer incidence while they bear 8% and 9% out of the global mortality and DALYs due to breast cancer. Our findings showed a wide range in the quality of breast cancer care, varying from 13.04 (Eastern Sub-Saharan Africa) to 96.58 (high-income Asia Pacific region) on the breast cancer QCI scale. A pivotal strategy in addressing the burden of breast cancer is the implementation of comprehensive management protocols, guaranteeing the feasibility and quality of cancer care. Population-level breast cancer screening programmes and improved treatment strategies have contributed to advancements in breast cancer care.^19 20^ However, communicable diseases have attracted the focus of the global health agenda for a long time and left cancer lower on the priority list, and the breast cancer prevention and care were not listed in the monitoring framework of UHC measurement.^21^ Low-income countries were markedly less likely than high-income countries to have national cancer control plans, limited funding for early detection programmes, and breast cancer referral systems. As breast cancer incidence grows rapidly and it is projected to account for 2 964 197 new cases and 1 046 512 deaths by 2040,^22^ with the majority occurring in low-income and middle-income countries, great concerns emerged and called for urgent attention and actions promoting the breast cancer care in these regions.

We observed a correlation between breast cancer QCI and social development levels, with low SDI regions achieving only 23.44% of the QCI levels observed in high-development regions, which has been reported in cervical cancer and gastric cancer previously.^23 24^ Socioeconomic situation could affect the availability of drugs, kits, radiation facility, pathology, oncology doctors and trained staff. For example, the density of radiotherapy units and radiotherapy utilisation was much lower in low- and middle-income countries than that in high-income countries.^25^ An estimation of the global distribution of operating theatres illustrated an averaged more than 14 per 100 000 population for high-income subregions while less than 2 per 100 000 population in low-income regions.^26^ The broken health system and inadequate financial protection also aggravated the inequality between countries in different social development levels. Average health insurance coverage was 7.9% in low-income countries, 27.3% in lower middle-income countries and 52.5% in upper middle-income countries.^27^ Then the high risk of catastrophic health expenditure would be a barrier to accessibility of high-quality breast cancer care.

The positive association between UHC and the breast cancer QCI underscores the pivotal role of UHC and resilient health systems in enhancing disease-specific care standards. We found a close correlation between UHC and quality of breast cancer care. Traditionally, the importance of quality of care in achieving UHC has been acknowledged. A high-quality healthcare system is defined as one ‘that optimises healthcare in a given context by consistently delivering care that improves or maintains health outcomes, by being valued and trusted by all people, and by responding to changing population needs’.^28^,^31^ We suggest that a resilient and proficient healthcare system, underpinned by UHC, is essential to enhance the quality of breast cancer care, with the β estimation reaching 0.89 (95% CI 0.74, 1.04) after adjusting for country-level SDI. Alongside the improvement of UHC and a strong health system, diagnostic and treatment service of breast cancer care could be more of accessibility, availability and affordability and lead to a reduced disease burden of breast cancer. In 2018, the WHO underscored the delivery of quality health services as a global imperative for UHC, identifying safety, effectiveness, efficiency, integration, equity, timeliness and people-centredness as key elements of healthcare quality.^32^ The Lancet Global Health Commission on High Quality Health Systems^33^ proposed a systems-based approach to improving quality within a robust enabling environment that fosters quality through leadership at various levels of the healthcare system.^34 35^

This study possesses various strengths. First, it undertook a systematic assessment of the status and disparities in regional and national quality of breast cancer care. Its findings may be a useful reference in formulating global strategies. Second, the study identified a robust association between UHC and the breast cancer QCI, underscoring the pressing need to strengthen healthcare systems to enhance the quality of care for this disease. However, the study also had certain limitations. The QCI index was constructed using the disease burden data with the initial concept that quality care will not only decrease the mortality rate of cancers, but also extend the life length (represented in decreasing YLLs), improve the life quality (represented in decreased DALYs and YLDs) and lead to more people living with the disease (represented in the prevalence of the breast cancer). While the QCI index capture the whole process of breast cancer care including diagnosis, treatment and rehabilitation, this index fails to measure the quality of breast cancer care process and care cascades. The evaluation of breast cancer care quality solely through the breast cancer burden fails to consider aspects such as the provision of care services and financial sustainability. Second, the study applied the global disease data from 1990 to 2019. Although not the latest estimation of the global disease burden, this study provided a unique observation of UHC in promoting the global breast cancer care.

## Conclusions

This study conducted a systematic evaluation of the present state and temporal trends in the global, regional and national quality of breast cancer care. Regional disparity existed regarding the quality of breast cancer care, especially in low-development countries. A robust and resilient health system was crucial for enhancing the quality of breast cancer care, highlighting the necessity of incorporating breast cancer care into the monitoring framework of UHC.

## supplementary material

10.1136/bmjph-2023-000863 online supplemental file 1

## Data Availability

Data are available in a public, open access repository.
