# Supplementary material for: Role of universal health coverage in improving quality of breast cancer care: an international comparison study
Source: BMJ Public Health. 2024 Sep 24;2(2):e000863. doi: 10.1136/bmjph-2023-000863 (PMC11816737; doi:10.1136/bmjph-2023-000863)
Supplement: online supplemental file 1 [file bmjph-2-2-s001.pdf]

**[Supplemental Material]**

# The Role of Universal Health Coverage in Improving Quality of Breast Cancer Care

**Supplemental Table 1.** The breast cancer quality of care index (QCI) by Global Burden of Disease (GBD) regions in 1990 and 2019.

**Supplemental Table 2.** The sensitivity analysis using the universal healthcare coverage index for years 2000, 2010 and 2015, and breast cancer quality of care index (QCI).

**Supplemental Figure 1.** The age pattern of breast cancer quality of care index (QCI), by regions defined by sociodemographic index (SDI).

**Supplemental Table 1. The breast cancer quality of care index (QCI) by Global Burden of Disease (GBD) regions in 1990 and 2019.**

| GBD regions | 1990 | 2019 | Percentage change (%) |
|---|---|---|---|
| Eastern Sub-Saharan Africa | 2.92 | 18.46 | 632.19 |
| Central Sub-Saharan Africa | 2.86 | 13.04 | 455.94 |
| Western Sub-Saharan Africa | 10.83 | 24.35 | 224.84 |
| South Asia | 17.2 | 36.09 | 209.83 |
| Andean Latin America | 29 | 58.39 | 201.34 |
| Southeast Asia | 28.43 | 53.36 | 187.69 |
| Tropical Latin America | 42.66 | 64.04 | 150.12 |
| North Africa and Middle East | 44.98 | 64.69 | 143.82 |
| East Asia | 57.31 | 81.3 | 141.86 |
| Central Latin America | 48.7 | 68.39 | 140.43 |
| Oceania | 19.35 | 27.01 | 139.59 |
| Southern Latin America | 52.8 | 69.69 | 131.99 |
| Southern Sub-Saharan Africa | 26.86 | 35.43 | 131.91 |
| Central Europe | 63.56 | 76.91 | 121 |
| Central Asia | 52.5 | 62.72 | 119.47 |
| Eastern Europe | 65.06 | 75.75 | 116.43 |
| Caribbean | 55.25 | 64.21 | 116.22 |
| Western Europe | 81.39 | 92.83 | 114.06 |
| Australasia | 84.93 | 94.82 | 111.64 |
| High-income North America | 86.71 | 94.35 | 108.81 |
| High-income Asia Pacific | 95.76 | 96.58 | 100.86 |

GBD, Global Burden of Disease; QCI, Quality of Care Index.

**Supplemental Table 2. The sensitivity analysis using the universal healthcare coverage index for years 2000, 2010 and 2015, and breast cancer quality of care index (QCI)**

| UHC index | Regression model | In 194 countries | In high and high-middle SDI regions | In middle, low-middle and low SDI regions |
|---|---|---|---|---|
| UHC 2000 | Univariable model | 1.30 (1.22, 1.37) | 0.81 (0.69, 0.93) | 1.35 (1.19, 1.50) |
| | Multivariable model | 0.72 (0.59, 0.86) | 0.58 (0.43, 0.73) | 0.88 (0.67, 1.09) |
| UHC 2010 | Univariable model | 1.49 (1.40, 1.57) | 1.11 (0.93, 1.29) | 1.30 (1.17, 1.43) |
| | Multivariable model | 0.87 (0.71, 1.02) | 0.76 (0.54, 0.99) | 0.92 (0.72, 1.12) |
| UHC 2015 | Univariable model | 1.52 (1.43, 1.61) | 1.18 (0.99, 1.37) | 1.29 (1.15, 1.42) |
| | Multivariable model | 0.85 (0.69, 1.00) | 0.82 (0.57, 1.07) | 0.88 (0.68, 1.08) |

QCI, quality of care index; SDI, sociodemographic index; UHC, universal healthcare coverage.

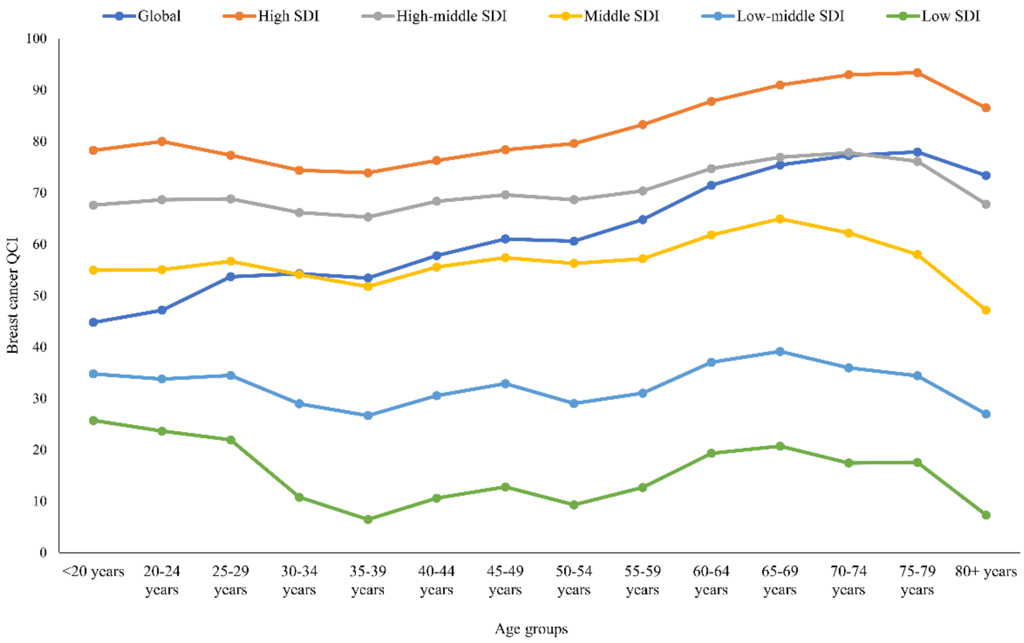


**Supplemental Figure 1. The age pattern of breast cancer quality of care index (QCI), by regions defined by sociodemographic index (SDI).**
